# A Series of microRNA in the Chromosome 14q32.2 Maternally Imprinted Region Related to Progression of Non-Alcoholic Fatty Liver Disease in a Mouse Model

**DOI:** 10.1371/journal.pone.0154676

**Published:** 2016-05-02

**Authors:** Kinya Okamoto, Masahiko Koda, Toshiaki Okamoto, Takumi Onoyama, Kenichi Miyoshi, Manabu Kishina, Jun Kato, Shiho Tokunaga, Taka-aki Sugihara, Yuichi Hara, Keisuke Hino, Yoshikazu Murawaki

**Affiliations:** 1 Second Department of Internal Medicine, Tottori University School of Medicine, Yonago, Tottori, Japan; 2 Department of Hepatology and Pancreatology, Kawasaki Medical School, Kurashiki, Okayama, Japan; Icahn School of Medicine at Mount Sinai, UNITED STATES

## Abstract

**Background & Aims:**

Simple steatosis (SS) and non-alcoholic steatohepatitis (NASH) are subtypes of non-alcoholic fatty liver disease (NAFLD), and the pathogenic differences between SS and NASH remain unclear. MicroRNAs (miRNAs) are endogenous, non-coding, short RNAs that regulate gene expression. The aim of this study was to use animal models and human samples to examine the relationship between miRNA expression profiles and each type of NAFLD (SS and NASH).

**Methods:**

DD Shionogi, Fatty Liver Shionogi (FLS) and FLS *ob/ob* mice were used as models for normal control, SS and NASH, respectively. Microarray analysis and real-time PCR were used to identify candidate NAFLD-related miRNAs. Human serum samples were used to examine the expression profiles of these candidate miRNAs in control subjects and patients with SS or NASH.

**Results:**

Fourteen miRNAs showed clear expression differences among liver tissues from SS, NASH, and control mice with good reproducibility. Among these NAFLD candidate miRNAs, seven showed similar expression patterns and were upregulated in both SS and NASH tissues; these seven candidate miRNAs mapped to an miRNA cluster in the 14q32.2 maternally imprinted region delineated by *delta-like homolog 1* and *type III iodothyronine deiodinase* (Dlk1-Dio3 mat). Software-based predictions indicated that the *transforming growth factor-β* pathway, *insulin like growth factor-1* and *5' adenosine monophosphate activated protein kinase* were potential targets of theses Dlk1-Dio3 mat NAFLD candidate miRNAs. In addition, serum samples from patients with SS or NASH differed markedly with regard to expression of the putative Dlk1-Dio3 mat miRNAs, and these differences accurately corresponded with NAFLD diagnosis.

**Conclusion:**

The expression profiles of seven miRNAs in 14q32.2 mat have high potential as biomarkers for NAFLD and for improving future research on the pathogenesis and treatment of NASH.

## Introduction

Non-alcoholic fatty liver disease (NAFLD) is increasing worldwide; NAFLD is defined by significant lipid deposition in hepatocytes (exceeding 5–10% of fat-laden hepatocytes were observed by light microscope) that is unrelated to excessive alcohol consumption [1]. NAFLD is a spectral disease. Currently, the most widely accepted mechanism for NAFLD progression is the “multiple parallel hit theory”, which is an expansion of the “two-hit theory” [2, 3]. The multiple-hit mechanism starts with the development of insulin resistance. Some life styles, including the excessive intake of dietary fructose can cause insulin resistance [4]. Insulin resistance leads to hyperinsulinemia, which upregulates hepatic *de novo* lipogenesis and adipose tissue lipolysis. These “primary hits” in hepatocytes increase susceptibility to multiple pathogenetic factors such as upregulation of pro-inflammatory cytokines and eicosanoids, Fas ligand and Toll-like receptor ligands; increase in reactive oxygen species (ROS); and altered production of adipokines [5]. Whole body organs such as adipose tissue, gut and gut microbiota also take part in the pathology [6, 7]. These factors promote hepatocyte apoptosis through mitochondrial dysfunction [8] and an endoplasmic reticulum stress reaction [9]. Continuous liver tissue injury progresses to liver fibrosis through activation of pro-fibrosis cytokines such as transforming growth factor ß (TGF-ß) [10]. All of these factors interact each other and drive a case of NAFLD toward NASH.

Nevertheless, the clinical status of any one NAFLD patient is classified broadly into one of just two categories, simple steatosis (SS) or non-alcoholic steatohepatitis (NASH) [11]. SS encompasses most of the NAFLD spectrum and is a benign condition.

NASH is the other end of NAFLD spectrum and defined as the combination of steatosis with lobular inflammation and hepatocyte ballooning; it can progress to liver fibrosis and result in cirrhosis and cancerous malignancies [11]. Different from SS, NASH is a life-threatening disease. In fact, a cohort study shows that 35% of NASH patients died during the 7.6-year follow-up period, but no SS patients died during the same period [12]. Considering the wide disease spectrum that results in significant prognosis differences among patients with NAFLD, the existence of some mechanisms that regulate one or more of these multiple-hit factors certainly exist.

MicroRNAs (miRNAs) are a class of endogenous, noncoding, small RNAs that regulate gene expression [13]. Mature miRNAs are introduced into RNA-induced silencing complexes (RISCs) [14]. A RISC bearing a miRNA usually binds to a partially complementary sequence within the 3’ UTR region of mRNAs and thereby either represses the translation or induces the degradation of those mRNA. Because base-pairing over just seven or eight bases of the miRNA seed region can elicit a miRNA-mediated effect, a single miRNA can regulate many target genes via mRNA regulation [15]. Owing to these features, miRNAs play an important role in many cellular processes including metabolism, inflammation, and fibrosis [16]. Accumulating evidence indicates that miRNAs are aberrantly expressed in metabolic tissues of obese animals, including humans and potentially contribute to the pathogenesis of obesity-associated complications [17, 18].

Recent evidence indicates that miRNAs contribute to the pathogenesis and progression of NAFLD both in animal models and human NAFLD patients [19–22]. For example, the expression levels of miR-29c, miR-34a, miR-155, and miR-200b in mouse model liver and miR-122 and miR-34a in human liver are suggested to be NASH development candidates. However, which miRNAs play important roles in the progression from SS to NASH remains unknown. The aims of the present study were 1) to clarify which miRNAs relate to NAFLD and NASH development by comparing mice, a mouse model of SS, and a mouse model of NASH, 2) to predict the potential target genes of NAFLD candidate miRNAs, and 3) to determine whether serum levels of human homologs of candidate miRNAs correlated with SS and NASH diagnoses and clinical features of NASH.

## Materials and Methods

### Ethics statement

This study was approved by the committee for ethics in medical experiments on animals and human subjects of the medical faculty of Tottori University (protocol No. 2374) and Kawasaki University (protocol No. 1814). The study was conducted in accordance with the declaration of Helsinki. Written informed consent was obtained from each patient before blood was collected. All animal experiments were carried out in accordance with the animal experimentation guidelines of Tottori University.

### Animal models

We used DD Shionogi (DS), Fatty Liver Shionogi Wild (FLS W) and FLS *ob/ob* mice as the normal control, the SS model and the NASH model, respectively. Male DS mice were provided by Riken bio-resource center through the national bio-resource project of Japan (Riken BRC No. 03706). Male FLS W and male FLS *ob/ob* mice were obtained from Shionogi research laboratories (Shiga, Japan). DS and FLS W are inbred strains established from the same outbred ddN colony [23]. DS shows neither liver abnormalities nor fatty liver changes. FLS W spontaneously develop chronic hepatic steatosis without obesity. FLS *ob/ob* was developed by transferring the spontaneous obesity mutation of the leptin gene: *Lepob* (commonly referred as *ob*) of the C57BL/6JWakShi (B6) *ob/ob* mouse into the genome of FLS W mice by backcross matings [24]. FLS *ob/ob* mice show hyperphagia, obesity, hyperlipidemia, and diabetes mellitus and have histologically severe steatosis and hepatic fibrosis; ultimately each FLS *ob/ob* mouse develops liver cirrhosis. FLS W and FLS *ob/ob* mice can be considered among the closest animal models to the human SS and NASH, respectively, because of the pathological differences between FLS W and FLS *ob/ob*. Specifically, FLS W mice lack the metabolic syndrome present in FLS *ob/ob* mice, and these metabolic differences seem to simulate human the NAFLD multiple parallel hit theory. Furthermore, FLS W and FLS *ob/ob* mice develop their characteristic pathological status without maintenance on a special diet feeding such as a methionine and choline deficient diet. Animals were housed in a room maintained at a controlled temperature of 24 ± 2°C under a 12-h light-dark cycle. Animals were given *ad libitum* access to water and standard pellet feed from birth. For each strain, five 24–week-old male mice were sacrificed under pentobarbital anesthesia and whole-blood was immediately collected from the right ventricle. Each liver was harvested and cut into about 200 mg pieces and fixed in 10% formalin for histological analysis or fresh-frozen in liquid nitrogen then stored at -80°C in the freezer until miRNA extraction. Hematoxylin-eosin staining and Sirius red staining were performed to assess pathological changes in liver tissue specimens.

### NAFLD candidate miRNA selection

**Fig 1:**
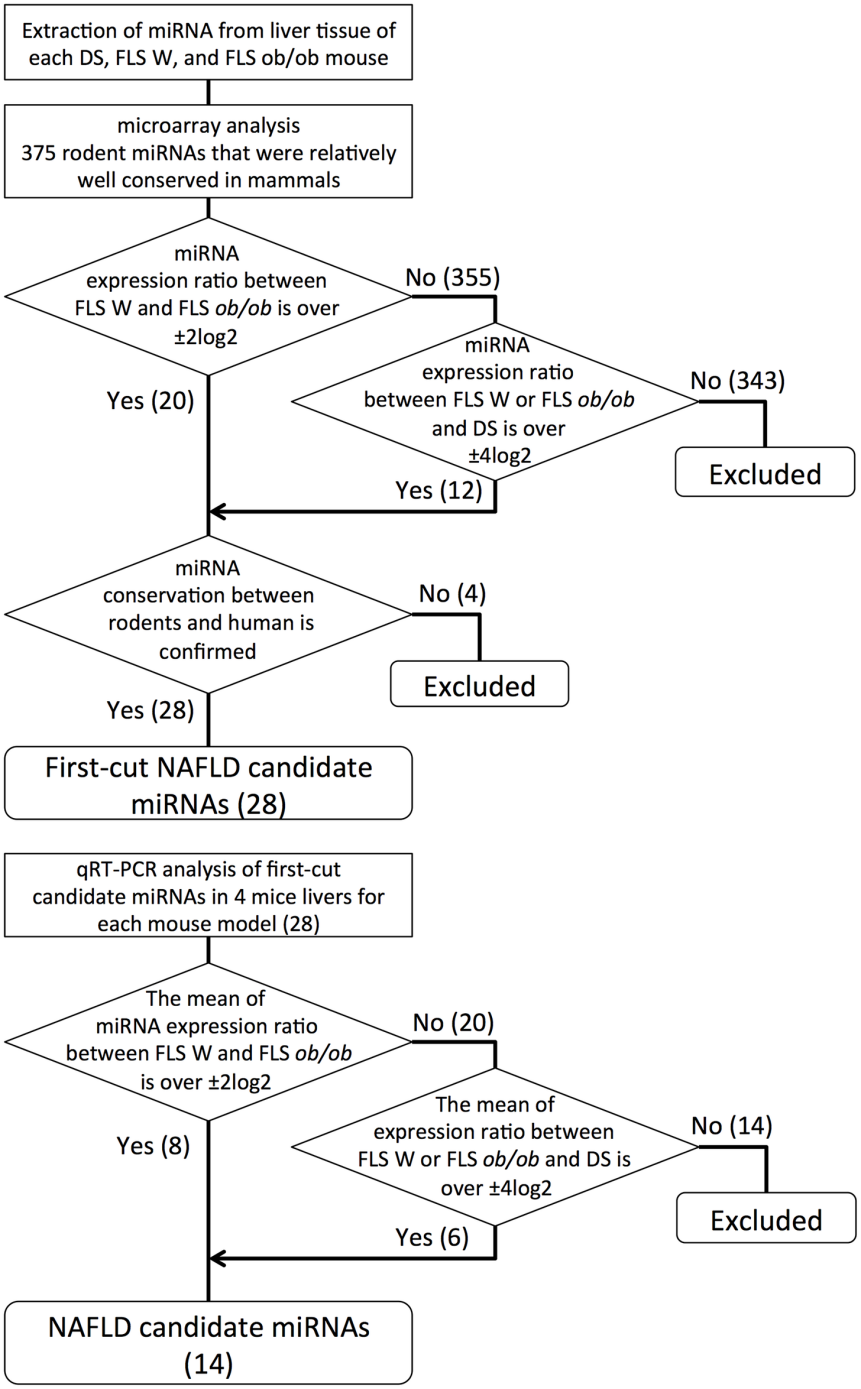
The protocol for selecting NAFLD candidate miRNA using mouse models of liver disease. The upper flowchart indicates the procedure for identifying first-cut candidates from microarray and phylogenetic analyses. The lower flowchart schematizes the reproducibility proofing for the first-cut candidates. The figures in parentheses indicate the number of miRNAs that fulfilled each selection criteria.

The procedure for NAFLD candidate miRNA selection is summarized in Fig 1. To identify NAFLD candidate miRNAs using the microarray data, we searched for miRNAs that had either of the following expression patterns: 1) a clear expression difference between the SS and NASH models; these candidates were predicted to relate to NAFLD progression or 2) clear difference in expression between the normal control and NAFLD (SS, NASH or both); these candidates were predicted to relate to NAFLD development. Therefore, we used two criteria: the candidate miRNA must show either 1) a normalized miRNA expression ratios that was greater than ±2log2 for FLS W and FLS *ob/ob* comparisons or 2) a ratio over ±4log2 for comparisons between FLS W or FLS *ob/ob* and DS.

To select candidates relevant to further study for human clinical conditions, the candidate miRNAs extracted from microarray were examined for sequence conservation between rodent and human. The microarray candidate sequences that were also certified as miRNAs in a human database miRBase were defined as first-cut NAFLD candidate miRNAs.

To reduce the risk of type 1 errors, we conducted quantitative real-time (qRT) PCR analysis of first-cut candidate miRNAs in four independent samples of mouse liver tissue for each of the mouse models and the normal controls. Those miRNAs with mean expression ratios based on qRT-PCR data that still satisfied criteria 1) or 2) were defined as NAFLD candidate miRNAs. Experimental details of the qRT-PCR and rodent-human sequence comparisons are described in more detail below.

### miRNA expression analysis in mouse liver

TrizolReagent (Life Technologies, Carlsbad, CA, USA) was used according to manufacturer’s protocols to extract total RNA from frozen mouse liver tissue. The quality of each total RNA sample was checked via an RNA Integrity Number (RIN), which is calculated by Agilent 2100 Bioanalyzer expert software (Agilent technologies, Santa Clara, CA, USA). Only high-quality RNA samples, those with an RIN greater than eight and A260/280 and A260/230 greater than 1.8, were used for microarray analysis. TaqMan^®^ Rodent microRNA A Card v3.0 (Applied Biosystems, Foster city, CA, USA) was used to assess the expression profiles of 375 miRNAs in mouse liver tissue. The probes were designed from miRNA sequence data in Sanger miRBase release 15. The qRT-PCR was used to validate the microarray results; miScript II reverse transcription kits, miScript SYBR^®^ Green PCR kits (Qiagen), and a Roche Lightcycler (Roche, Penzberg, Germany) were used for all qRT PCR assays. Small nuclear U6 RNA was used as an internal control.

### Microarray data deposition

The miRNA profiles, which were based on microarray data from the two mouse models, have been deposited in the NCBI Gene Expression Omnibus (http://www.ncbi.nlm.nih.gov/geo/) and are available under accession number GSE69670.

#### Analysis of miRNA conservation between rodent and human

The single sequence search function in the miRNA database miRBase version 20 (http://www.mirbase.org/) was used to assess conservation between rodent miRNA sequences and human miRNA sequences. SSEARCH was selected as the search method, and E-value cut-off was set to 0.05.

### Predicting the targets of the miRNAs

The putative target of the miRNAs were predicted using the web-driven software DIANA microT-CDS 5.0 (http://diana.cslab.ece.ntua.gr/) and Targetscan Human 6.0 (http://www.targetscan.org/). The threshold for the target prediction score in DIANA microT-CDS was set to 0.7. In Targetscan Human, the top 100 predicted targets as ranked by their aggregate probability of conserved targeting (PCT) were selected as target genes. DAVID 6.7 (http://david.abcc.ncifcrf.gov/) was used to perform gene ontology annotation; the Kyoto encyclopedia of genes and genomes (KEGG) were used for pathway enrichment analysis.

### Patient population and collection of blood samples

In all, 30 patients were enrolled in this study, either at Tottori University Hospital or Kawasaki University Hospital. Each of the three groups (patients with asymptomatic gallbladder stones (as disease controls), with SS, or with NASH) were comprised of 10 patients. The clinicopathological features of each patient group are shown in Table 1. All participants were Japanese and underwent continuous clinical follow up at the Tottori or Kawasaki University Hospital. Exclusion criteria included chronic hepatitis B or C virus infection, habitual alcohol consumption over 20 g / day, primary biliary cirrhosis, or autoimmune liver disease. Each SS or NASH patient underwent liver biopsy to confirm the diagnoses of SS and NASH, and for patients with NASH, the histological grade and stage of NASH was determined via the Brunt system [25]. Blood sample collection for serum miRNA and clinical blood tests were performed at same time point and within 3 months from liver biopsy. Blood samples were taken in fasted state. For each sample, blood serum was isolated by 4°C refrigerated centrifugation at 1500 x g for 10 minutes and then stored at -80°C in the freezer until use.

**Table 1:** Clinicopathological features of NAFLD and control patients.

				ANOVA p value		Bonferroni p value	
	NASH (n = 10)	SS (n = 10)	Control (n = 10)	NASH vs SS	NASH vs SS	NASH vs Control	SS vs Control
NASH Grade	2.0 ± 0	0.9 ± 0.6	-	0.00017*		-	-
NASH Stage	1.4 ± 1.0	0.6 ± 0.8	-	0.064		-	-
Age	51 ± 18	49 ± 11	62 ± 14		0.78	0.12	0.027
Gender (M/F)	5 / 5	6 / 4	5 / 5		0.52	>0.99	0.52
Height (cm)	160 ± 9	162 ± 7	163 ± 12		0.65	0.54	0.76
Body Weight (kg)	75 ± 15	70 ± 9	62 ± 11		0.39	0.048	0.12
Body Mass Index	29 ± 6	27 ± 3	23 ± 3		0.25	0.014*	0.044
T-Bil (mg/dL)	0.7 ± 0.2	1.0 ± 0.5	1.0 ± 0.4		0.15	0.080	0.89
AST (IU/L)	78 ± 40	25 ± 6	21 ± 5		0.0025*	0.0016*	0.12
ALT (IL/L)	141 ± 46	35 ± 15	19 ± 9		<0.0001*	<0.0001*	0.01
GGT (IU/L)	82 ± 38	69 ± 11	29 ± 15		0.73	0.0017*	0.27
T-Chol (mg/dL)	211 ± 29	209 ± 45	223 ± 38		0.94	0.41	0.47
LDL-Chol (mg/dL)	131 ± 28	119 ± 31	146 ± 27		0.43	0.38	0.17
HDL-Chol (mg/dL)	51 ± 13	51 ± 17	53 ± 28		0.99	0.94	0.94
TG (mg/dL)	171 ± 83	160 ± 56	119 ± 68		0.75	0.20	0.24
FBS (mg/dL)	121 ± 46	112 ± 20	96 ± 6		0.67	0.13	0.27
HgbA1c (%)	6.2 ± 1.1	5.7 ± 0.6	-	0.21		-	-
HOMA IR	4.0 ± 3.0	2.3 ± 0.4	-	0.16		-	-
Ferritin (ng/mL)	288 ± 255	144 ± 102	-	0.12		-	-

*: p < 0.05 in ANOVA and p < 0.016 in multiple comparisons (less than 0.05 / 3: adjusted by Bonferroni correction). Control: patients with asymptomatic gallbladder stone, T-Bil: total bilirubin, AST: alanine aminotransferase, ALT: aspartate aminotransferase, GGT: gamma glutamyl transferase T-Chol: total cholesterol, LDL-Chol: low-density lipoprotein cholesterol, HDL-Chol: high-density lipoprotein cholesterol, TG: triacylglycerol, FBS: fasting blood sugar, HOMA IR: homeostasis model assessment of insulin resistance.

### miRNA expression analysis with human serum

The miRNeasy serum/plasma kit (Qiagen, Venlo, Nederland) was used according to the manufacturer’s instructions to extract miRNAs from human serum samples. For each 200-μL serum sample, 1.6 x 10^8^ copies of *C*. *elegans* (Ce)-miR-39-1 (Qiagen) was used as non-mammal spike-in control and 1 μg bacteriophage MS2 RNA (Roche) was used as carrier RNA. The miScript II reverse transcription kit, the miScript SYBR^®^ Green PCR kit (Qiagen), and a Roche Lightcycler (Roche) were used for qRT-PCR amplification of serum miRNA.

### Hierarchical clustering of miRNA expression profiles

Cluster 3.0 software (http://www.eisenlab.org/eisen/) was used for hierarchical clustering of the miRNA expression profiles in human serum. Uncentered correlation and centroid linkage were selected as the similarity metric and clustering method, respectively. TreeView 1.60 (http://taxonomy.zoology.gla.ac.uk/rod/rod.html) was used to construct the cluster heat-map.

### Statistical Analysis

Statistical analysis was performed using software JMP 11.1 (SAS Institute Inc., Cary, NC, USA). Value data are expressed as the mean ± standard deviation. Statistical significance of differences between groups was determined using analysis of variance (ANOVA) and the Student's t test. Receiver operating characteristic (ROC) curve analysis was performed to assess the diagnostic accuracy of SS and NASH in human patients. Differences were considered statistically significant at p value < 0.05. In multiple comparisons, the p values were adjusted by Bonferroni correction to maintain the family-wise error rate.

## Results

### Histology of FLS W and FLS *ob/ob* liver tissue

**Fig 2:**
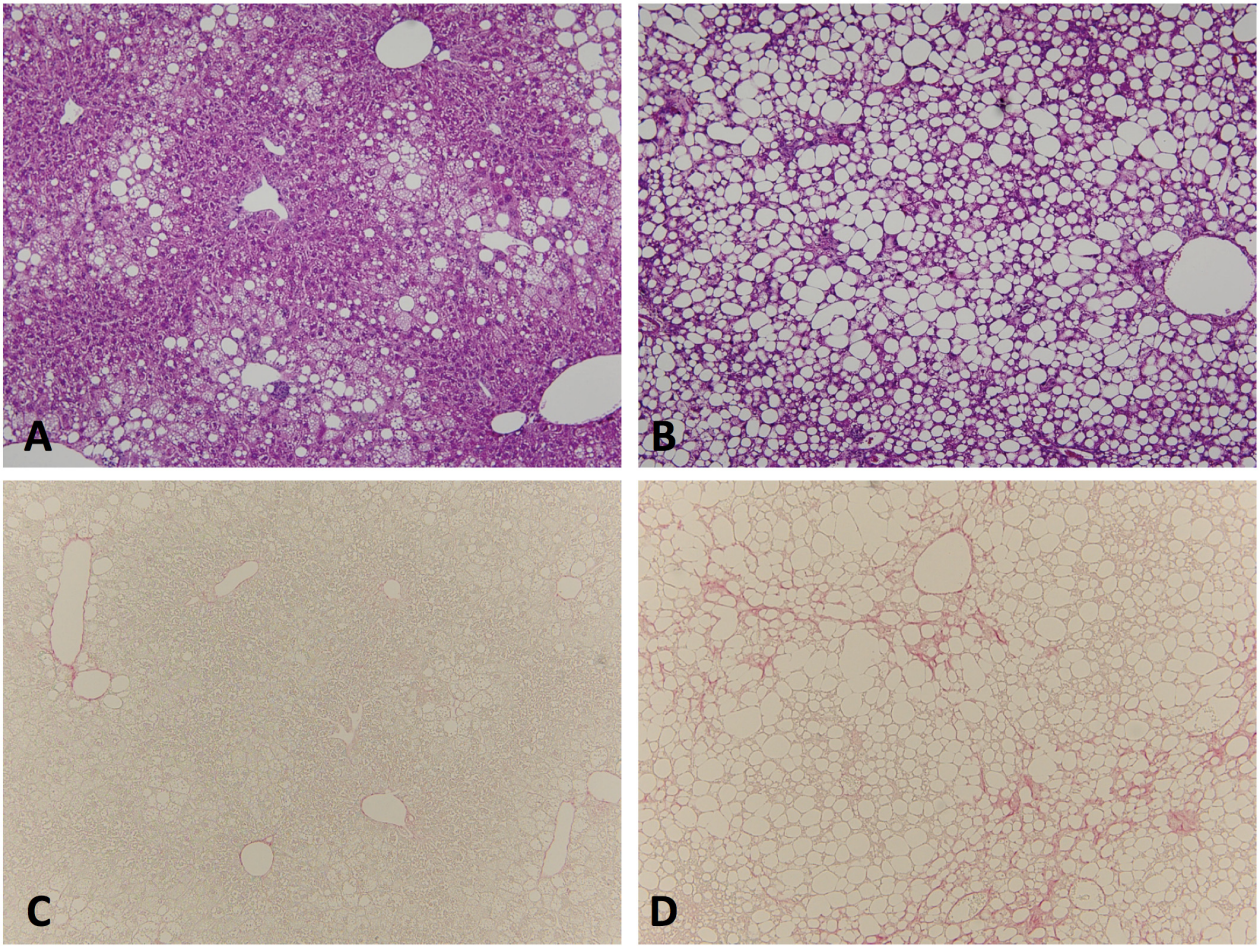
Hepatic histological findings in the fatty liver Shionogi Wild (FLS W) mice and FLS *ob/ob* mice. A and B: HE staining (magnification: x 100) in FLS W and FLS *ob/ob* mice at 24 weeks of age, respectively. C and D: Sirius red staining (magnification: x100) in FLS W and FLS *ob/ob* mice at 24 weeks of age, respectively.

To assess NAFLD progression in each mouse model, histological examinations of liver sections were performed (Fig 2). FLS W liver tissue exhibited moderate simple steatosis. FLS *ob/ob* liver tissue exhibited several features associated with NASH, including severe fat deposition, hepatocytes ballooning, infiltration of inflammatory cells, and fibrosis development.

### miRNA microarray analysis in mouse models of liver disease

The microarray analysis showed that 32 miRNAs fulfilled the preliminary selection criteria for NAFLD candidate miRNAs (S1 Table). We then confirmed that 28 of these 32 miRNAs were well conserved between rodents and human and identified as first-cut candidates (S2 Table). The four rodent-specific miRNAs—mmu-miR-351, mmu-miR-434, mmu-miR-467a, and mmu-miR-682—were excluded from further studies.

### qRT PCR validation of miRNA candidates

**Fig 3:**
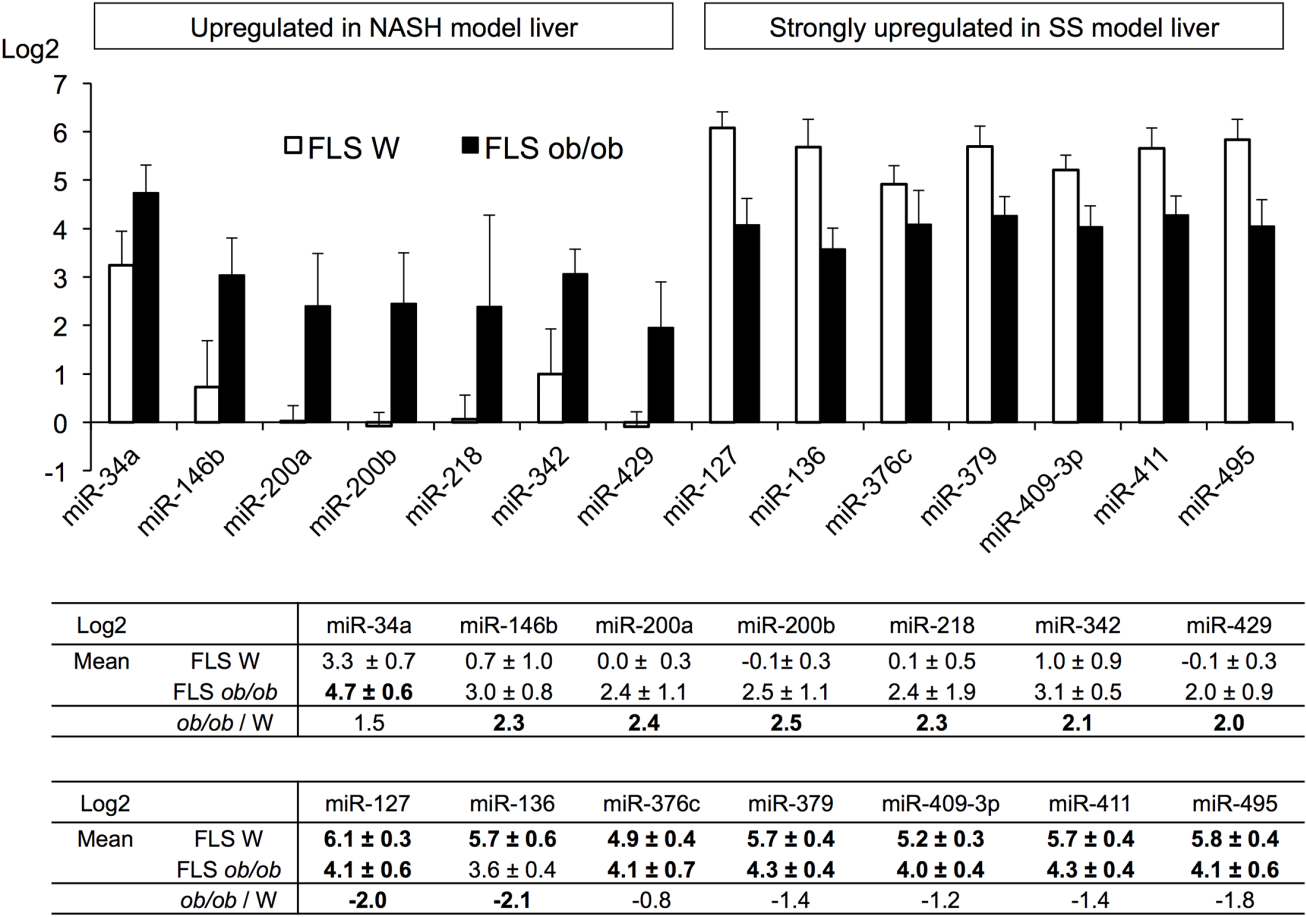
Relative expression of candidate miRNAs in liver tissue from mouse models of SS or NASH. Microarray data were used to identify these candidates, and qRT-PCR from four specimens was used to determine the validity of the microarray findings. All candidate miRNA qRT-PCR data were normalized to U6 RNA data, and fold changes were calculated relative to data from normal control DS liver.

The reproducibility validation of the microarray analysis was performed by qRT-PCR. The mean expression ratio for each of 14 miRNAs fulfilled our criteria for candidate miRNAs. We defined these 14 miRNAs as candidate NAFLD miRNAs (Fig 3). The expression patterns of putative NAFLD miRNAs seemed to form two groups. Seven of these miRNAs (miR-34a, -146b, -200a, -200b, -218, -342, and -429) were strongly upregulated in FLS *ob/ob* mice relative to FLS W mice. The other seven (miR-127, -136, -376c, -379, -409-3p, -411, and -495) were upregulated in both FLS W and FLS *ob/ob* mice relative to control mice; however, expression of these seven was higher in the FLS W mice than in FLS *ob/ob* mice.

Most of the candidate miRNAs that were strongly upregulated in FLS *ob/ob* mice only: miR-34a, -146b, and miR-200 family (miR-200a, -200b, and -429) had already been identified as NASH-promoting miRNAs [19–22]. Furthermore, the roles of these miRNAs in NASH pathology have been partially indicated previously [26].

### Putative miRNA clusters in the human genome

Many miRNAs are distributed into miRNA clusters in the human genome [27]. Based on previous studies, we have perceived that a part of NAFLD candidate miRNAs (miR-200a, -200b, and -429) is in the same cluster [28, 29]. miR-200a, -200b, and -429 are coded in the same primary miRNA on chromosome 1p36. Indeed, the expression patterns of these three candidate miRNAs in NASH model mouse closely resembled each other (Fig 3). To examine the other relationships between the candidate NAFLD miRNAs and miRNA clusters, we used miRBase ver. 20 to determine whether the putative NAFLD miRNAs were clustered (Table 2).

**Table 2:** Genomic location of the putative miRNAs in human.

Putative miRNA	Location
miR-34a	1p36.22
miR-146b	10q24.32
miR-200a, -200b, -429	1p36.33
miR-218	4p15.31
miR-127^†^, -136^†^, -342, -376c^†^, -379^†^, -409-3p^†^, -411^†^, -495^†^	14q32.2

^†^ Located in the maternally imprinted gene cluster delineated by Dlk1 and Dio3

Strikingly, eight of the candidate NAFLD miRNAs were in close proximity to each other at genome position 14q32.2. Furthermore, among these eight miRNAs, seven (miR-127, -136, -376c, -379, -409-3p, -411, and -495) were each upregulated in both FLS W and FLS *ob/ob* liver and mapped to the same maternally imprinted gene cluster delineated by the *delta-like homolog 1* gene and the *type III iodothyronine deiodinase* gene (Dlk1-Dio3 mat) [30]. Expression of this miRNA cluster is regulated by an intergenic, germline-derived, differentially methylated region located 200 kbp upstream from the miRNA cluster [30, 31].

### Software-based predictions of miRNA target genes

In the subsequent experiments, we focused on the newly discovered NAFLD candidate miRNAs in the Dlk1-Dio3 mat cluster. We predicted the potential target proteins for each candidate miRNA (Table 3).

**Table 3:** Software predicted candidate Dlk1-Dio3 mat miRNA target genes.

	Ontology Predicted Targets
miR-127	**TGF-ßR1**
miR-136	CD28, Fas-L, PI3K
miR-376c	CXCL1, 5, 16, IFNa6, IL-1RAP, IL-2, PDGFRa, ß, PI3K, **Smad4, Smurf1, TGF-ßR1**, TNFSF4
miR-379	**AMPK**, Bcl-2, Collagen IV, HGF, **IGF1,** IGF1R, PDGF, PG-F2R, PTEN, **Smad4, Smurf1,** SREBP1, **TGF-ßR1**
miR-409-3p	**AMPK,** Collagen IV, **IGF1,** IL-2, PKC, **Smad**2,**4, TGF-ßR1**, 2
miR-411	**AMPK,** Bcl-2, GSK3b, HGF, **IGF1,** IGF1R, IL-1a, MMP-7, PDGF, PTEN, **Smad4, Smurf1**
miR-495	**AMPK** Bcl-2, Caspase 8, CD8b, Collagen IV, FAS, GSK3B, Hexokinase2, HGF, **IGF1**, IGFR1,IL-1ß, IL-1R, IL-6, IRS1,2, JAK3, LDL-R, Leptin, Leptin R, MMP-14, PDGFa, PDGFRa, PI3K, PKC, PPARa, PTEN, **Smad**2, **4**, **Smurf1,** TGF-ß1, **TGF-ßR1**, R2

The boldface genes are frequently predicted as targets for more than four of these seven miRNAs. The standard nomenclature for each of the abbreviations used in this table is listed in S3 Table.

Some genes were frequently identified as targets of the candidate Dlk1-Dio3 mat miRNAs. For the seven candidate Dlk1-Dio3 mat miRNAs, *transforming growth factor beta receptor 1* (TGF-βR1: 5 of 7), *small phenotype / mothers against decapentaplegic 4* (Smad4: 5 of 7), *Smad ubiquitin regulatory factor1* (Smurf1: 4 of 7), *Insulin like growth factor 1* (IGF1: 4 of 7) and *5' adenosine monophosphate activated protein kinase* (AMPK: 4 of 7) were each frequently identified as putative targets.

### Expression of NAFLD candidate miRNAs in Dlk1-Dio3 mat in human serum

**Fig 4:**
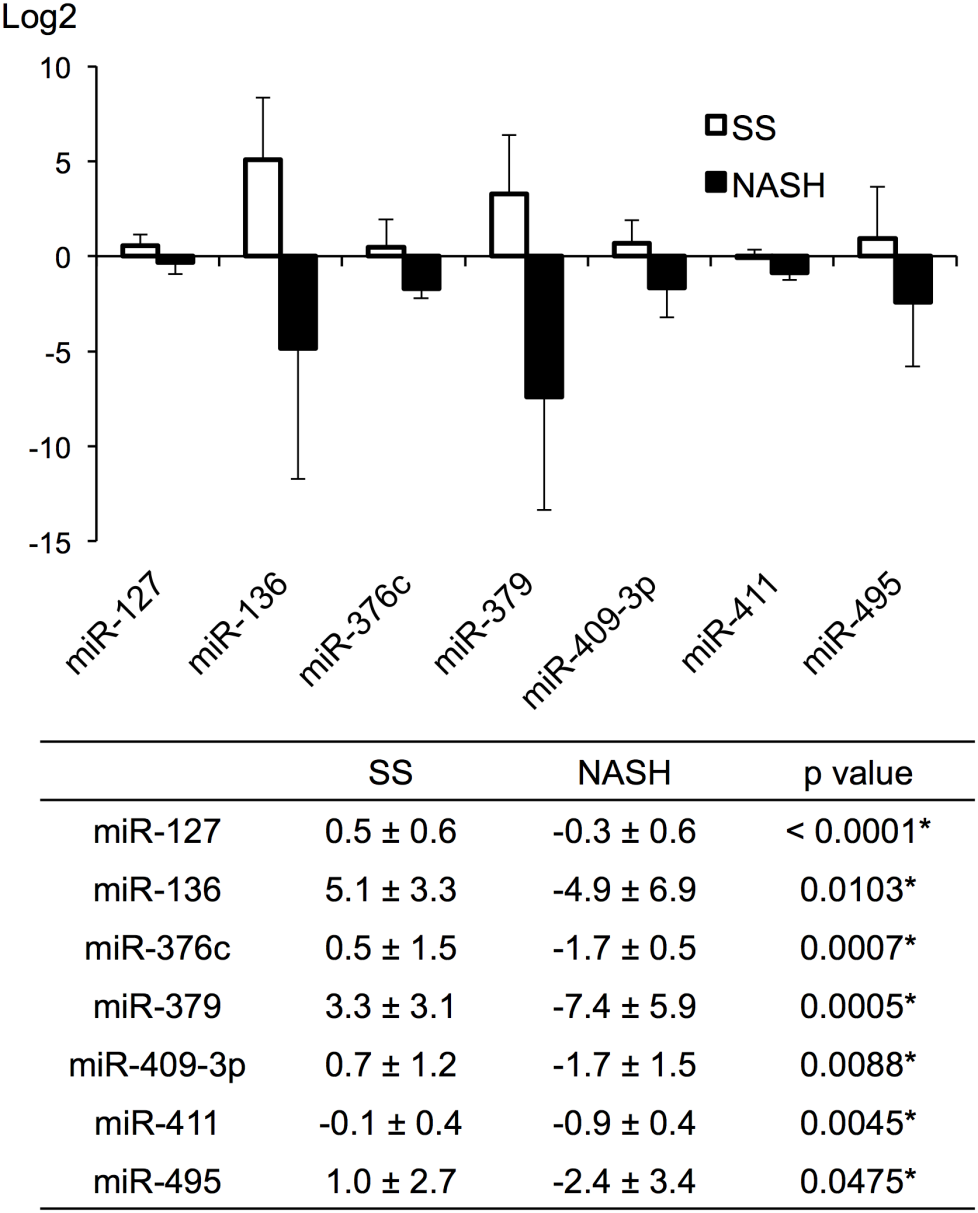
Putative miRNA expression levels in human SS and NASH patients. Normalized relative to Ce-miR-39-1; values represent fold difference relative to the normal control.

**Fig 5:**
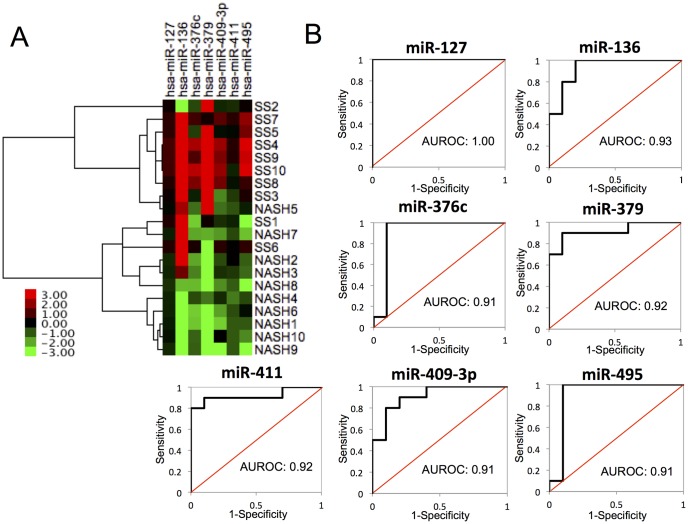
**(A) Hierarchical clustering among Dlk1-Dio3 mat NAFLD candidate miRNAs between SS and NASH patients.** Normalized relative to Ce-miR-39-1; values represent fold difference relative to the normal control. **(B) The ROC curves of Dlk1-Dio3 mat NAFLD candidate miRNAs.**

As a preliminary study of the clinical relevance of these miRNAs, we examined the serum levels of the respective human homolog of each Dlk1-Dio3 mat candidate NAFLD miRNA using serum samples from control, SS, or NASH patients. The expression patterns of Dlk1-Dio3 mat candidate miRNAs in human serum samples differed from the patterns in the mouse models of liver disease (Fig 4). However, the hierarchical clustering analysis showed that Dlk1-Dio3 mat candidate NAFLD miRNA expression profile in human serum clearly differed between human SS and NASH samples (Fig 5A). Furthermore, for each of these miRNAs, the area under the ROC curve (AUROC) was 0.91 to 1.00 for the SS or NASH diagnostic group (Fig 5B); these results indicated that each of the Dlk1-Dio3 mat NAFLD candidate miRNAs provided good diagnosis accuracy with regard to distinguishing between SS and NASH for NAFLD patients.

### Associations between serum levels of candidate miRNAs and clinicopathological features of NASH patients

We analyzed correlations between clinicopathological features and serum levels of Dlk1-Dio3 mat NAFLD candidate miRNAs in NASH patients.

In the histological analysis of human liver biopsy specimens, NASH grade for each patient with NASH was grade 2 (moderate) adventitiously (Table 1). Among the group of patients with NASH, stages of fibrosis stages ranged from 0 to 3. The serum level of Dlk1-Dio3 mat candidates and fibrosis progression were not significantly associated in any pair-wise relationship (S1 Fig).

We also examined the relationships between serum levels of candidate miRNAs and clinical blood test values of NASH patients. Linear regression analysis showed some associations between serum levels of the NAFLD candidate miRNAs in Dlk1-Dio3 mat miRNA and clinical parameters of NASH patients; however, these associations were not significant after Bonferroni adjustment (S2 Fig).

## Discussion

### Seven miRNAs in Dlk1-Dio3 mat are newly identified as NAFLD candidate miRNAs

In the present study, we identified a novel set of NAFLD candidate miRNAs—miR-127, -136, -376c, -379, -409-3p, -411, and -495—that all mapped to the same miRNA cluster in the human Dlk1-Dio3 mat region. These miRNAs shared the same promoter region and showed similar and specific expression patterns in the liver tissue of each NAFLD mouse model.

miRNAs in Dlk1-Dio3 mat region had been identified as candidate for various diseases including cancer, psychiatric illnesses and alcoholism [32–34]. However, to our knowledge, no published reports indicate clear relationships between miRNAs in the Dlk1-Dio3 mat cluster and NAFLD.

A few published studies show relationships between miRNAs in the Dlk1-Dio3 mat region and NAFLD-related metabolic functions. For example, Labialle et al. reports that part of Dlk1-Dio3 mat region (the miR-379 / miR-410 cluster), which included five of the candidate NAFLD miRNAs (miR-376c, -379, -409, -411, and -495), affects energy metabolism including gluconeogenesis in neonatal mouse [35]; selective ablation of the miR-379 / miR-410 cluster causes lethal blood hypoglycemia. The finding of Labialle et al. seems to strengthen our hypothesis that hepatic overexpression of our candidate NAFLD miRNAs from Dlk1-Dio3 mat may affect hepatic energy metabolism. Additionally, patients with a congenital disease called maternal uniparental disomy for chromosome 14 (upd(14)mat) show some symptoms similar to Prader-Willi syndrome. Patients with upd(14)mat exhibit characteristic weight gain in early childhood that results in truncal obesity [36]. Theoretically, the maternally imprinted genes on chromosome 14 including the entire Dlk1-Dio3 mat miRNA cluster is overexpressed in upd(14)mat. The Dlk1-Dio3 mat candidate NAFLD miRNAs may play some roles in obesity progression.

Interestingly, the miRNAs in the Dlk1-Dio3 mat region have high sequence similarities to each other [37]. Indeed, TGFβ-R1, Smad4, Smurf1, IGF1 and AMPK were frequently identified specific target genes of each Dlk1-Dio3 mat miRNA.

TGF-β is a potent pro-fibrosis cytokine, and it plays multiple roles including in apoptosis and inflammation [38]. The TGF-β receptor, TGFβ-R1, is expressed on various intrahepatic cells including hepatocytes, endothelial cells, and stellate cells; this receptor transfers the TGF-β signal to intracellular mediators [38]. Smad4 transduces the TGF-β signal to target genes in the nuclear genome [38]. In contrast, Smurf1 inhibits the TGF-β signaling pathway [39]. The Dlk1-Dio3 mat candidate NAFLD miRNAs probably influence NAFLD pathology by modifying TGF-β signaling mediators.

IGF-1 is an insulin-like, anabolic hormone that improves insulin sensitivity and accelerates lipid oxidation and lipolysis [40]. IGF-1 is mainly secreted from hepatocytes, and circulating IGF-1 levels reflect hepatic IGF-1 expression [40]. Serum IGF-1 level is significantly lower in both SS and NASH patients [41]. Strong upregulation of the candidate NAFLD miRNAs that target IGF-1 probably relates to NAFLD development. In addition, our IGF-1-related candidate NAFLD miRNAs were moderately but significantly more downregulated in liver tissue from NASH than from SS model mice; the mean expression was 2.7 ± 1.2 log2-fold lower in the NASH mice (p values < 0.001). IGF-1 expression levels in both serum and liver tissue are not correlated to liver fibrosis progression in NASH patients [42]. The partial downregulation of candidate NAFLD miRNAs that target IGF-1 in NASH mice may relate to the paradoxical IGF-1 expression in NASH patients.

The sets of predicted targets of the NAFLD candidate miRNAs in Dlk1-Dio3 mat overlapped, and this finding suggested that these candidate miRNAs may affect specific liver functions in a cooperative manner.

AMPK is a key sensor of energy status in mammalian cells, and it plays a critical role in the repression of glycolysis, lipogenesis, and the promotion of fatty acid oxidation in the liver [43]. However, previous findings in animal models show that total AMPK expression level does not differ significantly between NASH and normal livers [43, 44]. AMPK is a heterotrimer comprising one catalytic subunit α (α1 or α2) and two regulatory subunits ß (ß1 or ß2) and γ (γ1, γ2 or γ3). Detailed KEDD analysis predicted that miR-146b, -379, and -411 could interfere with either catalytic subunit α1 or α2. However, the affinity between each of these three miRNAs and each of the catalytic isoforms (α1 and α2) were not assessed in the KEDD analysis. miR-409-3p was predicted to target α2 only. These four candidate miRNAs may play some role in regulating expression level of each subunit to some degree.

The Dlk1-Dio3 region contains 54 miRNAs and is one of the largest known miRNA clusters in the human genome [32]. We examined the expression levels of the other candidate miRNAs in Dlk1-Dio3 mat. For example, miR-134, -337, -381, and -412 were included in our microarray analysis. Moreover, miR-134 and miR-337 were strongly upregulated in livers from FLS W mice, and we selected these miRNAs for the first round of predictions (S1 Table). However, the qRT-PCR data from these four miRNAs did not fit the selection criteria. Signals from the miR-412 probe were not detected in FLS W samples, and miR-381 signals were not detected in FLS W or FLS *ob/ob* samples. Other miRNAs in the Dlk1-Dio3 mat were not identified in our microarray screen. Further detailed analysis of the relationship between the Dlk1-Dio3 mat miRNA cluster and NAFLD pathology is expected.

### miR-218 and miR-342 were also newly predicted candidate NAFLD miRNAs based on mouse models

Additionally, we are the first to suggest that miR-218 and -342 are candidate NAFLD miRNAs. miR-218 was upregulated in liver from NASH model mice and miR-218 is a well studied cancer-preventive miRNA [45]. Moreover, a recent study showed that miR-218 targets adiponectin receptor 2 (AdipoR2) in cultured hepatoma cells, HepG2, and attenuates the adiponectin signal [46]. Adiponectin is a NASH-preventing adipokine and indeed hepatic AdipoR2 expression is downregulated in NASH patients [47]. miR-218 may have a role in NASH progression via AdipoR2 downregulation.

The miR-342 gene is located in 14q23.2, but not in the Dlk1-Dio3 mat region [30], and miR-342 expression is coupled to the host gene Ena/Vasp-like protein [48]. Indeed, the miR-342 expression pattern differed from those of the Dlk1-Dio3 mat candidate miRNAs. Further studies will be needed to clarify the function of miR-218 and miR-342 in NAFLD pathology.

### Discrepancy between liver tissue miRNA levels and serum miRNA levels

In our study, miRNA expression levels often differed between mouse liver tissues and human serum samples. Notably, the liver and serum miRNA expression profiles were examined in different species, and this type of discrepancy between serum and liver is evident in many previously published miRNA including studies of NASH [49, 50]. There are two factors that may drive the differences between organ tissues and serum with regard to miRNA levels. First, serum miRNA expression is a sum total of secretion from all organs throughout the body. Notably, NAFLD pathology can be explained in the context of multiple, complex organ reactions. miRNA expression in other NAFLD-related organs (e.g., adipose tissue, pancreas and gut) may influence serum miRNA levels. For example, over expression of miR-127, a Dlk1-Dio3 mat NAFLD candidate, in pancreas islet cells suppresses insulin secretion and causes glucose intolerance [51]; additionally, miR-342 and miR-379 are upregulated in white adipose tissue of obese mice [52]. Moreover, selective inclusion of miRNAs into exosomes may cause different tissues to exhibit different levels of the same miRNA. Exosomes play a major role in cellular miRNA secretion into body fluids [53]. Exosomes contain proteins and nuclear acids (including miRNAs) and protect these miRNA from degradation by RNases and urinal secretion. Recent studies clearly demonstrate that sorting functions affect miRNA incorporation into exosomes [53]. However, even with the existence of intervening mechanisms, serum profiles of our candidate miRNAs differed distinctly between SS and NASH patients and these differences conveyed high diagnosis accuracies. Furthermore, the levels of several candidate NAFLD miRNAs were significantly correlated with clinicopathological features of NASH patients. The candidate miRNAs identified here may become very meaningful and useful to clinical diagnosis of human NAFLD and to treatment of this disease spectrum.

### Study limitations

Our study had some limitations because of the sample size and the study design.

To identify candidate NAFLD miRNAs, we adjusted the threshold ratio of miRNA expression to ratios between the FLS W and FLS *ob/ob* groups of > ±2log2 or between the FLS W or FLS group and the control group of > ±4log2. Based on these criteria, we selected nearly 10% of all measured miRNAs (32 candidate miRNAs / 375 scanned miRNAs) as candidate NAFLD-related miRNAs. Nevertheless, this threshold might have been too high, and we may have missed important miRNAs. For example, Cheung et al. suggests that miR-122 expression in liver tissue relates to NASH development and the log2 ratio to normal expression was -0.29 [19], which was similar to our result (-0.66 and -0.28 in FLS W and FLS *ob/ob* liver, respectively).

We used software programs to predict the target genes of the candidate miRNAs. This method is commonly used; however, it involves the risk of missing some real targets because the software is designed to assess the relative strength of partial sequence complementary between mRNA and miRNA. Ontology selection was used to select putative targets that might be relevant to cell functions. However, ontology selection can only identify proteins whose functions have been identified. Notably, our understanding of the detailed mechanisms that lead to NAFLD development and progression to NASH is still developing, and new insights are being made regularly.

Moreover, we did not substantiate that any NAFLD candidate miRNA actually interfered with any of the predicted target genes *in vivo* (mouse model livers) or *in vitro*, like the direct binding experiments. Complex intracellular regulatory networks influence tissue-specific function of miRNAs [54]; therefore future study is needed to assess whether the predicted targets are actual targets of these miRNAs.

The number of patient in this study was small; there were only 10 people in each group. Consequently, the statistical powers of the human serum data were relatively limited.

Our findings from NAFLD mouse models were not really confirmed by miRNA expression profiling in human liver tissue. A parallel examination of microarrays of human liver samples would have enhanced the confidence of NAFLD candidate miRNAs. However, we have not examined miRNA expression profiling in human liver tissues primarily because we did not have access to liver tissue specimens from normal control subjects due to ethical considerations.

Owing to these limitations of our study, larger human population-based studies are warranted to confirm and extend our findings.

## Conclusion

An inclusive analysis of miRNA expression in liver tissue from NAFLD mouse models revealed a group of candidate NAFLD miRNA that included seven newly discovered candidates located in the same miRNA cluster within Dlk1-Dio3 mat on chromosome 14. TGF-β signaling mediators and IGF-1 were predicted as the targets of most candidate NAFLD miRNAs in Dlk1-Dio3 mat.

Bases on preliminary study of serum from patients with NAFLD, the candidate miRNAs in Dlk1-Dio3 mat region had specific expression patterns that provided high diagnostic accuracy with regard to SS and NASH patients.

To identify with confidence associations with highly complex and interactive miRNA effects, future longitudinal studies with greater sample size will be necessary.

## Supporting Information

S1 Fig Dlk1-Dio3 mat candidate miRNA expression for different NASH stages. Normalized relative to Ce-miR-39-1; values represent fold difference relative to the normal control.
(TIF)

S2 Fig Linear regression analysis between serum Dlk1-Dio3 mat candidate miRNA expression levels and clinical features of patients with NASH. Only relationships with a p-value < 0.05 are indicated. p < 0.007 (less than 0.05 / 7: adjusted by Bonferroni correction) is significant.
(TIF)

S1 Table Microarray-based predictions of relative expression of candidate miRNAs in liver from mouse models of SS and NASH. Candidate miRNA level was normalized to U6 RNA level. Criteria used to select candidate miRNAs were as follows: 1) normalized miRNA expression ratios (FLS W: FLS *ob/ob* expression) more than ±2log2 and/or 2) FLS W: DS or FLS: DS ratios more than ±4log2.
(DOCX)

S2 Table Analysis of sequence conservation between rodent NAFLD candidate miRNAs and corresponding human miRNAs. The single sequence search function of miRBase was used. The search method was SSEARCH, and the E-value cut off was set to 0.05. X indicates no corresponding miRNA was found in the human genome.
(DOCX)

S3 Table Candidate miRNAs and the corresponding target genes with abbreviations. (DOCX)
