# Supplementary figures and images for: A Series of microRNA in the Chromosome 14q32.2 Maternally Imprinted Region Related to Progression of Non-Alcoholic Fatty Liver Disease in a Mouse Model
Source: PLoS One. 2016 May 2;11(5):e0154676. doi: 10.1371/journal.pone.0154676 (PMC4852931; doi:10.1371/journal.pone.0154676)

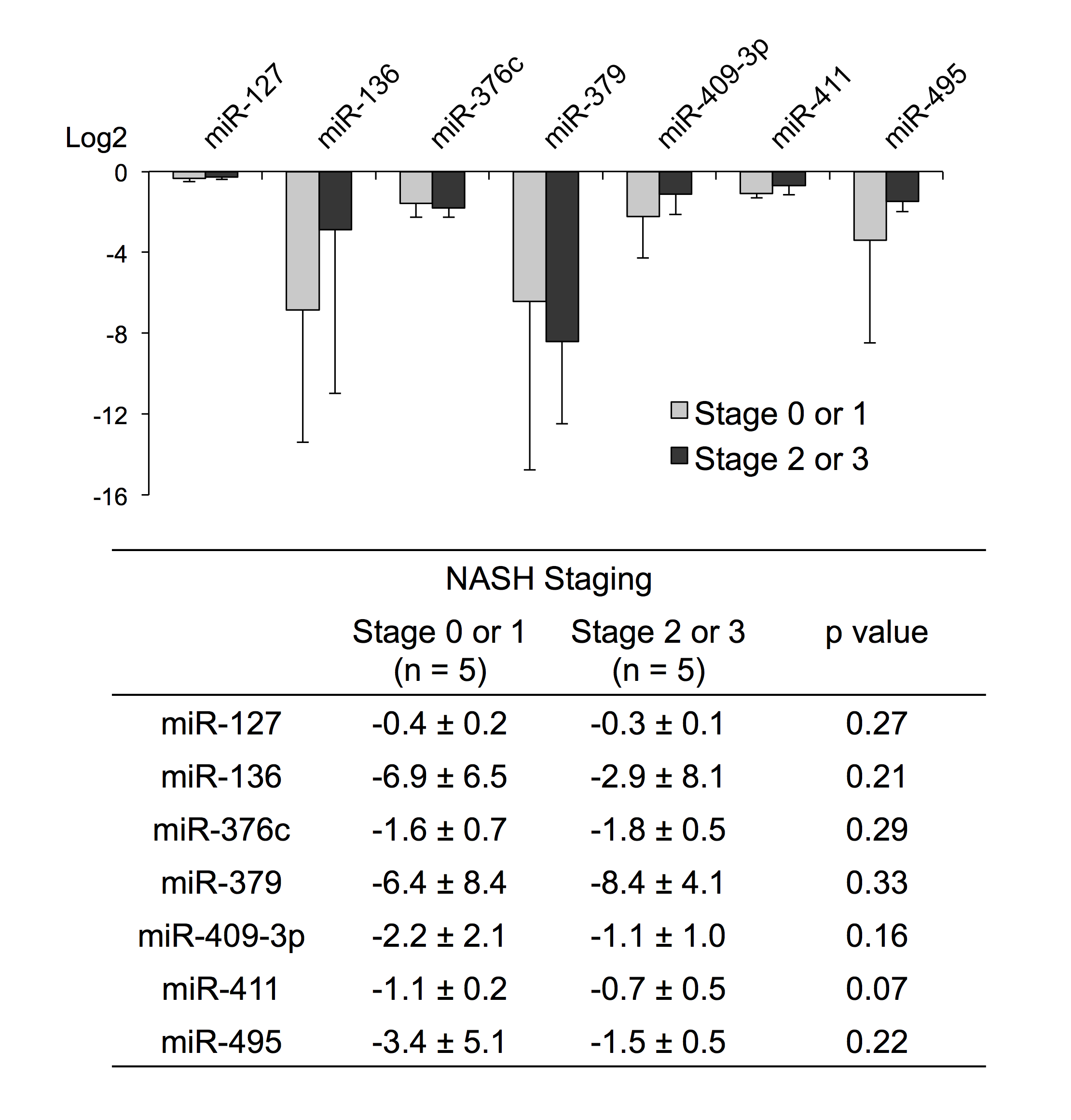

Supplement: S1 Fig — Normalized relative to Ce-miR-39-1; values represent fold difference relative to the normal control. (TIF) [file pone.0154676.s001.tif]

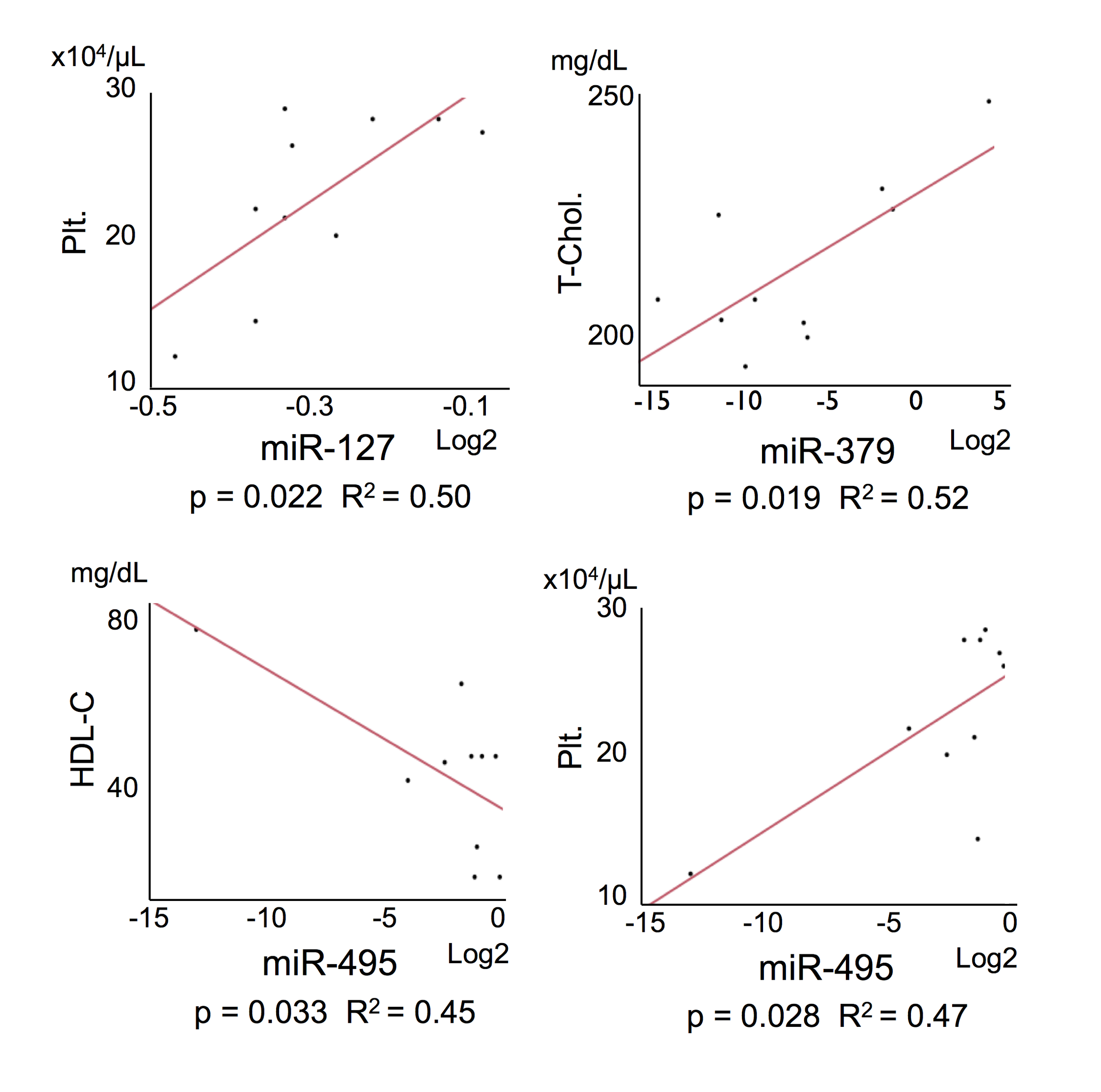

Supplement: S2 Fig — Only relationships with a p-value < 0.05 are indicated. p < 0.007 (less than 0.05 / 7: adjusted by Bonferroni correction) is significant. (TIF) [file pone.0154676.s002.tif]
