# Supplementary material for: A Series of microRNA in the Chromosome 14q32.2 Maternally Imprinted Region Related to Progression of Non-Alcoholic Fatty Liver Disease in a Mouse Model
Source: PLoS One. 2016 May 2;11(5):e0154676. doi: 10.1371/journal.pone.0154676 (PMC4852931; doi:10.1371/journal.pone.0154676)
Supplement: S1 Table — Candidate miRNA level was normalized to U6 RNA level. Criteria used to select candidate miRNAs were as follows: 1) normalized miRNA expression ratios (FLS W: FLS ob/ob expression) more than ±2log2 and/or 2) FLS W: DS or FLS: DS ratios more than ±4log2. (DOCX) [file pone.0154676.s003.docx]

| Candidate  miRNAs | log2  FLS W / DS | log2  FLS *ob/ob* / DS | log2  FLS W / FLS *ob/ob* |
| --- | --- | --- | --- |
| rno-miR-1 | -2.90 | 1.28 | **-4.18** |
| mmu-miR-10b | **4.65** | **4.26** | 0.39 |
| mmu-miR-27a | **4.04** | 3.62 | 0.42 |
| mmu-miR-32 | **4.24** | **4.60** | -0.36 |
| mmu-miR-34a | 3.77 | **4.75** | -0.98 |
| mmu-miR-107 | 0.02 | 2.48 | **-2.46** |
| mmu-miR-127 | **5.56** | 3.31 | **2.25** |
| mmu-miR-134 | **5.53** | 2.77 | **2.76** |
| mmu-miR-136 | **4.37** | 1.83 | **2.54** |
| mmu-miR-146b | **4.77** | 3.93 | 0.85 |
| mmu-miR-148b | 3.92 | **4.25** | -0.33 |
| mmu-miR-181c | **4.68** | **4.16** | 0.52 |
| mmu-miR-182 | 2.45 | **4.35** | -1.90 |
| mmu-miR-200a | 0.73 | 2.83 | **-2.11** |
| mmu-miR-200b | 0.88 | 3.15 | **-2.28** |
| mmu-miR-214 | 3.33 | **4.03** | -0.70 |
| mmu-miR-218 | -0.10 | 2.21 | **-2.31** |
| mmu-miR-337-3p | **5.81** | 2.78 | **3.02** |
| mmu-miR-342-3p | **7.02** | **5.45** | 1.57 |
| mmu-miR-345-3p | -2.22 | 0.19 | **-2.41** |
| mmu-miR-351 | **4.42** | 2.15 | **2.28** |
| mmu-miR-376b | **5.63** | 2.92 | **2.71** |
| mmu-miR-376c | **6.62** | 3.31 | **3.31** |
| mmu-miR-379 | **5.45** | 3.17 | **2.29** |
| mmu-miR-409-3p | **7.86** | 3.73 | **4.14** |
| mmu-miR-411 | **7.10** | 3.56 | **3.54** |
| mmu-miR-429 | 1.36 | 3.56 | **-2.20** |
| mmu-miR-434-3p | 0.09 | -3.56 | **3.64** |
| mmu-miR-434-5p | 0.08 | -3.56 | **3.65** |
| mmu-miR-467a | **4.35** | 3.30 | 1.05 |
| mmu-miR-495 | **5.98** | 3.30 | **2.69** |
| mmu-miR-511 | 1.12 | -1.62 | **2.73** |
| mmu-miR-682 | **4.14** | 3.31 | 0.82 |

**S1 Table: Microarray-based predictions of relative expression of candidate miRNAs in liver from mouse models of SS and NASH.** Candidate miRNA level was normalized to U6 RNA level. Criteria used to select candidate miRNAs were as follows: 1) normalized miRNA expression ratios (FLS W: FLS *ob/ob* expression) more than ±2log2 and/or 2) FLS W : DS or FLS : DS ratios more than ±4log2.
