# Supplementary material for: A Series of microRNA in the Chromosome 14q32.2 Maternally Imprinted Region Related to Progression of Non-Alcoholic Fatty Liver Disease in a Mouse Model
Source: PLoS One. 2016 May 2;11(5):e0154676. doi: 10.1371/journal.pone.0154676 (PMC4852931; doi:10.1371/journal.pone.0154676)
Supplement: S2 Table — The single sequence search function of miRBase was used. The search method was SSEARCH, and the E-value cut off was set to 0.05. X indicates no corresponding miRNA was found in the human genome. (DOCX) [file pone.0154676.s004.docx]

| Candidate miRNAs in  Rodent | Corresponding miRNAs of  Homo sapiens | E-value |
| --- | --- | --- |
| rno-miR-1 | hsa-miR-1-2 | 6.4e-18 |
| mmu-miR-10b | hsa-miR-10b | 1.5e-15 |
| mmu-miR-27a | hsa-miR-27a | 2.3e-17 |
| mmu-miR-32 | hsa-miR-32 | 1.8e-18 |
| mmu-miR-34a | hsa-miR-34a | 3.6e-20 |
| mmu-miR-107 | hsa-miR-107 | 2.5e-21 |
| mmu-miR-127 | hsa-miR-127 | 1.2e-19 |
| mmu-miR-134 | hsa-miR-134 | 3.0e-18 |
| mmu-miR-136 | hsa-miR-136 | 1.5e-15 |
| mmu-miR-146b | hsa-miR-146b | 6.0e-16 |
| mmu-miR-148b | hsa-miR-148b | 1.1e-24 |
| mmu-miR-181c | hsa-miR-181c | 5.1e-24 |
| mmu-miR-182 | hsa-miR-182 | 2.7e-18 |
| mmu-miR-200a | hsa-miR-200a | 3.2e-22 |
| mmu-miR-200b | hsa-miR-200b | 3.2e-17 |
| mmu-miR-214 | hsa-miR-214 | 5.8e-26 |
| mmu-miR-218 | hsa-miR-218 | 1.4e-28 |
| mmu-miR-337 | hsa-miR-337 | 4.0e-20 |
| mmu-miR-342 | hsa-miR-342 | 2.6e-27 |
| mmu-miR-345 | hsa-miR-345 | 2.4e-15 |
| mmu-miR-351 | X | 5.5 (hsa-miR-3934) |
| mmu-miR-376b | hsa-miR-376b | 4.7e-18 |
| mmu-miR-376c | hsa-miR-376c | 2.4e-10 |
| mmu-miR-379 | hsa-miR-379 | 2.3e-16 |
| mmu-miR-409-3p | hsa-miR-409-3p | 3.6e-5 |
| mmu-miR-411 | hsa-miR-411 | 1.9e-22 |
| mmu-miR-429 | hsa-miR-429 | 1.4e-31 |
| mmu-miR-434 | X | No Match |
| mmu-miR-467a | X | No Match |
| mmu-miR-495 | hsa-miR-495 | 3.4e-10 |
| mmu-miR-511 | hsa-miR-511 | 1.3e-14 |
| mmu-miR-682 | X | No Match |

**S2 Table: Analysis of sequence conservation between rodent NAFLD candidate miRNAs and corresponding human miRNAs**. The single sequence search function of miRBase was used. The search method was SSEARCH, and the E-value cut off was set to 0.05. X indicates no corresponding miRNA was found in the human genome.
