# Supplementary material for: A Series of microRNA in the Chromosome 14q32.2 Maternally Imprinted Region Related to Progression of Non-Alcoholic Fatty Liver Disease in a Mouse Model
Source: PLoS One. 2016 May 2;11(5):e0154676. doi: 10.1371/journal.pone.0154676 (PMC4852931; doi:10.1371/journal.pone.0154676)
Supplement: S3 Table — (DOCX) [file pone.0154676.s005.docx]

| Abbreviation | Gene name |
| --- | --- |
| AdipoR | *Adiponectin receptor* |
| AMPK | *AMP activated protein kinase* |
| ACSL | *Long-chain acyl-CoA synthetase* |
| Bcl-2 | *B cell lymphoma 2* |
| CD | *Cluster of differentiation* |
| CXCL | *C-X-C motif ligand* |
| EGFR | *Epidermal growth factor receptor* |
| Fas L | *Fas ligand* |
| GSK3β | *Glycogen synthase kinase 3 beta* |
| G6P | *Glucose 6-phosphate* |
| HGF | *Hepatocyte growth factor* |
| HLADRB1 | *Human leukocyte antigen DR beta 1* |
| IFNα | *Interferon alpha* |
| IGF | *Insulin-like growth factor* |
| IGFR | *Insulin-like growth factor receptor* |
| IL | *Interleukin* |
| IL-1R | *Interleukin 1 receptor* |
| IL-1RAP | *Interleukin 1 receptor accessory protein* |
| IRAK | *Interleukin 1 receptor associated kinase* |
| IRS | *Insulin receptor substrate* |
| JAK | *Janus kinase* |
| LDLR | *Low-density lipoprotein receptor* |
| LeptinR | *Leptin receptor* |
| MAPK | *Mitogen-activated protein kinase* |
| MMP | *Matrix metalloproteinase* |
| PDGF | *Platelet-derived growth factor* |
| PDGFR | *Platelet-derived growth factor receptor* |
| PGF2R | *Prostaglandin F2 receptor* |
| PI3K | *Phosphoinositide 3-kinase* |
| PKC | *Protein kinase C* |
| PTEN | *Phosphatase and tensin homolog* |
| Smad | *Small phenotype mothers against decapentaplegic* |
| Smurf | *Smad ubiquitin regulatory factor* |
| SREBP | *Sterol regulatory element binding protein* |
| TGFβ | *Transforming growth factor beta* |
| TGFβR | *Transforming growth factor beta receptor* |
| TLR | *Toll-like receptor* |
| TNFSF | *Tumor necrosis factor superfamily member* |
| TRAF | *Tumor necrosis factor receptor associated factor* |
| UGT | *Uridine diphosphate galactose galactosyltransferase* |

**S3 Table: Candidate miRNAs and the corresponding target genes with abbreviations.**
